# Challenges in using patient involvement principles in substance use treatment

**DOI:** 10.1080/17482631.2023.2223424

**Published:** 2023-06-13

**Authors:** Njål Herman Eikeng Sterri, Jan. H. Rosenvinge, Gunn Pettersen

**Affiliations:** a Department of Health and Care Science, Faculty of Health Sciences, University of Tromsø, Norway; b Faculty of Health Sciences, VID Specialized University, Bergen, Norway; c Department of Psychology, Faculty of Health Sciences, University of Tromsø, Norway

**Keywords:** Patient involvement, Substance use treatment, Substance use disorder, Qualitative Research, Experiences

## Abstract

**Background:**

Health professionals are responsible for implementing patient involvement (PI) in the choice of treatment approach. Previous studies within the field of substance use disorder (SUD) treatment have shown positive patient experiences with PI. However, little is known about challenges experienced by health professionals in converting principles of PI into clinical practice.

**Aims:**

To explore challenges with PI in the treatment of SUD.

**Method:**

Five health professionals working in a Norwegian institution for inpatient treatment of SUD were included and took part in a semi-structured interview. Data were analysed using a systematic text condensation approach.

**Results:**

PI in SUD was perceived as challenging due to conceptual unclarities as well as treatment dilemmas that may challenge the notion of PI as a universal and unified ideological foundation of substance use treatment.

**Conclusions:**

The findings point to a need to critically examine the PI concept and to take a flexible approach in adjusting PI principles to good clinical practice. A framework is launched, allowing the reported challenges in implementing PI in clinical practice to be accepted, acknowledged, and recognized by clinicians as well as by administrators and heads of clinical units.

## Introduction

The importance of enabling patients to take an active role in their care is universally acknowledged (Goodhew et al., 2019; Tambuyzer et al., 2014). Notably, in the treatment of substance use disorders (SUD) patient involvement (PI) has been strongly advocated to achieve treatment goals, patient satisfaction, and patient cooperation (Brener et al., 2009; Patterson et al., 2009; Rance & Treloar, 2015), and thereby a favourable treatment outcome. Patients’ positive experiences of PI have been shown in a systematic review (Goodhew et al., 2019), and have been related to a decrease in substance use and criminal-justice problems (Fischer & Neale, 2008). PI has been generally acknowledged and outlined in many national legislations with respect to principles for how treatments and services should be organized and delivered. Nevertheless, previous studies (Fischer et al., 2008; Jansen & Hanssen, 2017; Rise et al., 2013; Sharp et al., 2021; Wenaas et al., 2021), and a systematic review (Bee et al., 2015), have shown that PI as a concept may be difficult to conceive for both health professionals and patients, and hence, there is a need for research that may guide how to convert general PI-principles to clinical practice (Jørgensen & Rendtorff, 2018; King, 2011). Conceptual and “translational” challenges may exacerbate in the treatment of SUD due to complications elicited by patients’ lack of insight and treatment compliance resulting in conflicting views about treatment plans and needs (Bee et al., 2015; Fischer & Neale, 2008; Fischer et al., 2008; Goodhew et al., 2019). Furthermore, needs voiced by patients with SUD may collide with legal restrictions, professional, ethical standards, or clinical evidence (Bee et al., 2015; King, 2011). Health professionals are responsible for involving patients in treatment, and hence their views and interpretations of PI are essential to contribute to a more precise conceptual understanding of PI than what has been noted in the field (Croft & Beresford, 1990). Moreover, challenges perceived by health professionals dedicated to implement PI in the treatment of SUD have been incomprehensively explored (Goodhew et al., 2019) and thus the aim of the present study was to explore such challenges.

## Method

### Context and participants

We approached a Norwegian specialized in-patient unit offering treatment varying between 3 to 12 months for patients with SUD or other addictive disorders. Core treatment elements were PI, and medication-assisted treatment that were run by interprofessional teams. Eligible to the study were health professionals with at least two years of clinical experience from the unit prior to the current study. Three males and two females aged 35–55 years, and with a professional background as a nurse or social worker were approached, and all of them consented to participate.

### Data collection and analysis

A semi-structured interview guide consisting was developed based on previous research findings and clinical experience. The interviews were conducted one time and started with the overall question; “How will you describe the contents and purpose of PI in SUD treatment?”. Follow-up questions probed into what kind of challenges with PI that the informants perceived, and why. All interviews were conducted digitally, audiotaped, and transcribed by the first author (NHES).

The interviews were analysed by a four-step inductive systematic text condensation approach (Malterud, 2012). In step one, the interviews were read through to establish an overall impression while searching broadly for recurring themes. In step two, meaningful entities were identified using line-by-line induction in N`Vivo 12 (QSR International Pty Ltd.). The third step consisted of abstracting meaning and condensing knowledge from the codes, and in the final step, the abstracted knowledge was recontextualised.

The first author (NHES) conducted the primary data analysis. To strengthen the validity and trustworthiness of the study all authors cooperated in validating the analysis, *i.e.*, discussing the coding, the categorization, the findings, and the interpretations until agreement was reached. According to the principles outlined by Malterud (2012) the final categories were reached through a process moving back and forth between the transcripts, the findings, the literature, and relevant theory to secure that the constructed descriptions were grounded in the empirical data. The authors had no relationship to the participants prior to the study.

### Ethics

The ethical standards laid down by the revised (2008) Helsinki Declaration were followed, and the study was approved by the Norwegian Centre for Research Data (NSD) (approval no. 360722). Hence, the study rested on the principles of written, informed consent and the option to withdraw from the study unconditionally until all data were de-identified. To ensure rigour and transparency, the consolidated criteria for reporting qualitative research (COREQ) (Tong et al., 2007) were followed.

## Results

### Conceptual unclarities

The participants were familiar with and aware of the overall purpose of PI and, *i.e.*, to promote patients’ empowerment, and self-worth through equity in the decision-making about their treatment plan. Nevertheless, they were confused about the actual meaning of PI, presenting conceptual unclarities regarding the PI term and its purpose in SUD treatment. Andrew, a social worker who had years of clinical experience, said:

“I don´t know how to describe patient involvement easily. It’s quite comprehensive, but to me, it’s about letting patients control their treatment plans. By control, I mean making decisions about what they want to achieve in treatment and how to achieve it. It is important to enable the patient to make choices in the treatment process; otherwise, how will they learn to take responsibility for their choices?”

Ann, a psychiatric nurse with years of clinical experience, shared this perception of PI:

”The concept is hard to define. To me, PI entails equity in decision-making regarding choices that will affect the patient’s life after treatment. Ensuring PI can be tricky, as no patient is alike regarding preferences, abilities and needs. Therefore, I guess how we ensure PI varies from one patient to another, trying to make the best out of every situation.”

The participants described two concurrent approaches to unravel the meaning and purpose of PI as a concept. James, a social worker with years of clinical experience, said:

”PI is about involving patients in treatment decisions to take charge of their recovery process. However, that does not mean that the patient solely makes treatment decisions. After all, there is a reason that they need help in mastering their addiction. Nevertheless, their voices need to be heard and taken into consideration, and for that, we have guidelines and tools, such as a feedback systems and treatment plans, which let patients’ voices systematically be heard and make it easier for us to adjust treatment to their needs and preferences.”

Joel, an experienced social worker, said:

“To me, PI is about trusting patients to make good choices and giving them the responsibility to do so. It is about involving patients in daily clinical encounters because patients’ needs and motivation are transient and fluctuating in nature, and as health professionals we must support them when needed. Sometimes, that means letting the patients decide whether to prioritise meaningful or social activities that goes beyond our treatment plans. We must trust them and not be too rigid to conform with treatment plans or house rules. In this way, we can let the patients form treatment content and purpose, and that counters everything you can do with PI tools. Even though it can be difficult to know when to restrict PI when patients don’t comply, listen, or lack insight into their addiction”.

In the first approach, PI was conceptualized by involving patients in treatment decisions through feedback tools and treatment plans. This approach allowed health professionals to follow the guidelines but was experienced as not giving patients real influence over their treatment. In the second approach, PI was conceptualized more directly through actively involving patients in daily clinical encounters based on patient needs and preferences.

### Treatment dilemmas

Three kinds of dilemmas were repeatedly mentioned. The first dilemma was to grant applications for a leave of absence to inpatients with a history of destructive behaviours. One may raise the risk of relapse, but on the other hand, restricting PI by not approving the application could deprive patients of experiencing the consequences of their actions. Ann said:

”If I perceive a patient to be in a vulnerable situation, for example, where the risk of a relapse is high, and the patients’ need for a leave of absence is strong, involving the patient in making treatment decisions can be very difficult. I cannot help but think that I’ll be partly responsible if the patient overdoses while on leave, but how can they learn from their choices if we deprive them of making them?”

Sharing these views, Petra, an experienced nurse, said:

”Granting patients a leave of absence can be very hard when I perceive the patient to be in a rather unstable phase in terms of motivation and substance use. It requires a lot of trust to involve patients in making these decisions because the consequences can be fatal. My professional opinion obviously conflicts with the patients’ needs, and that makes these situations very hard to solve.”

The second dilemma was how to respond to patients’ demands for medication when such a demand was judged as reflecting the SUD or a dysfunctional coping with abstinence-elicited distress. Petra said:

”When I strongly disagree with a patient’s perceived need for medication, such as benzodiazepines, patient involvement can be hard to ensure. My experience tells me that many patients use medication as a coping strategy to handle feelings of distress, making it challenging to involve them in decisions. If I comply with the patient’s perceived needs, to what degree do I help them? And if I don’t, to what extent do I restrict their right to be involved in treatment decisions?”

Moreover, this dilemma was also described by Ann, saying:

”From my experience, when patients have trouble sleeping or have had a bad day and just want the day to end, some want sleeping pills, such as Z-hypnotics. My professional judgment tells me that such medication is not a good solution, and I try to explain my thoughts to them. Yet, for some this solution is what they are used to, and that makes it hard to involve them in the decision.”

Complying with the demands for medication could ensure PI, but at the cost of giving in to unreasonable and destructive needs possible upholding addiction and a poor coping strategy. This placed the informants obliged to implement PI in a professional, ethical dilemma.

Finally, a reported dilemma was how to handle patients’ consistent demands for exemptions from mandatory collective activities and group therapy for rest and privacy reasons. Informants described it as difficult to allow exemptions to ensure PI, thereby depriving patients of necessary treatment. Petra said:

”Treatment consists of certain mandatory activities that patients are expected to partake in. What role does PI play if patients don’t want to partake? And to what extent should a patient’s perceived needs outweigh SUD interventions? Naturally, basic needs should be considered, and the patient’s voice should be heard. However, the patients are in treatment for a reason, and that reason is that their choices have not always served them well. If I let patients do what they want instead of participating in SUD interventions, do I ultimately help them or harm them?”

The informants also voiced a suspicion that demands for treatment exemptions might reflect patients’ treatment resistance or other avoidance strategies. James said:

“The balance between the individual and the collective is tough to find when trying to involve patients in treatment. There are patients with specific needs, such as an increased need for rest. If I allow the patient to be exempted from partaking, other patients often feel discriminated against. How to choose whose needs to meet and how to prioritise them is therefore quite hard”.

Although the three examples of difficult situations to handle are well known in the treatment of SUD, they were reported as dilemmas considering the mandatory obligation to ensure PI and the absence of clear guidelines to implement PI.

## Discussion

The present study explored how health professionals experienced challenges in using PI in the treatment of SUD. Three key findings should be highlighted.

*First*, the fact that informants experienced PI as conceptually ambiguous in nature aligns with a recent systematic review (Ocloo et al., 2021), depicting PI suffering from terminological confusions and no overarching theoretical framework. Other findings also indicate that PI is a problematic ideal to realize due to vagueness of aim and content (Jansen & Hanssen, 2017). A consistent framework may thus be needed in response to pleas in the literature (Jørgensen & Rendtorff, 2018; King, 2011), to close the gap between “theory” and clinical practice.

*Secondly*, in addressing challenges, the fact that health professionals had a mixed approach resulting in variations in how PI was practised may reflect that the use of tools to involve patients may overstate a theoretical approach to PI, thereby possibly limiting real patient influence. By contrast, direct involvement in daily clinical encounters seemed to focus on a practical aspect of PI, but this was challenging in clinical practice due to a vast amount of responsibility and individual assessments.

*Thirdly*, many dilemmas in the treatment of patients with SUD are well-known, notably how to handle patients’ requests that were deemed not to align with good clinical practice or the inpatient treatment programme. Adding a set of PI principles and concepts that were experienced as ambiguous and unclear enhanced clinical dilemmas through the discrepancy between theoretical positioning and clinical challenges and the fact that the health professionals were obliged to operate in accordance with the principles of PI. Our findings align with the few existing studies reporting on challenges experienced by health professionals (Brekke et al., 2018; Jansen & Hanssen, 2017).

The present study provides insight into practitioners’ experiences with challenges that may arise when providing SUD treatment according to PI principles, a phenomenon that has been incomprehensively explored (Bee et al., 2015; Goodhew et al., 2019). A recent systematic review (Tong et al., 2007), has pointed to health professionals as keys to PI as a pathway to equity and empowerment and resolving health professionals’ experience of PI as a challenge is essential to achieve such a key position, indicating the value of implementing a more consistent and overarching framework. The present study highlights that health care professionals working with SUD patients need to reflect on treatment dilemmas and thereby add practical meaning to key concepts of PI. Such reflections may depart from at least two queries; 1) the conceptual understanding of PI underpinning dilemma situations, and 2) understanding patients’ needs or demands against clinical experience, evidence-based practice, fellow patients, and the overall treatment structures. These queries may provide a useful framework to accomplish the need to critically examine the PI concept from ethical, clinical, and practical perspectives, and to take a flexible approach to adjust PI principles to patients’ individual needs. However, such a framework must be supported and implemented by heads of SUD-treatment units and clinical institutional leadership in general.

### Strengths and limitations

A previous systematic review (Ocloo et al., 2021), have shown how attitudes among personnel may represent a barrier to implementing PI. To our knowledge, the present study is original as being the first one exploring PI challenges in SUD treatment reported by health care professionals highly dedicated to implementing PI. Hence, it is less likely that our findings were flawed by negative attitudes towards adhering to PI principles in clinical practice. While the sample size could have been larger, the trustworthiness and transparency of our findings were based on the rigour (Whittemore et al., 2001), in which the rich, in-depth data were collected and analysed. Furthermore, the consolidated criteria for reporting qualitative research (COREQ) (Tong et al., 2007). using co-researchers strengthens credibility and in this study the research team provided the possibility to ongoing critical reflections and interpretations of data (Bryman, 2016). The participants had been educated and trained in many regions of the country. However, they were recruited to this study from only one of the regions and whether this recruiting might have limited data transferability remains unsettled, awaiting future studies from other clinical contexts that deliver SUD treatment based on PI.

## Conclusion

The findings point to a need to critically examine the PI concept and to take a flexible approach to adjust PI principles to good clinical practice. A framework is launched, allowing the reported challenges in implementing PI in clinical practice to be accepted, acknowledged, and recognized by clinicians as well as by administrators and heads of clinical units.
